# A Dynamic Precision Evaluation Method for the Star Sensor in the Stellar-Inertial Navigation System

**DOI:** 10.1038/s41598-017-04061-5

**Published:** 2017-06-28

**Authors:** Jiazhen Lu, Chaohua Lei, Yanqiang Yang

**Affiliations:** The Science and Technology on Inertial Laboratory, School of Instrumentation Science and Opto-electronics Engineering, Beijing University of Aeronautics and Astronautics, Beijing, 100191 China

## Abstract

Integrating the advantages of INS (inertial navigation system) and the star sensor, the stellar-inertial navigation system has been used for a wide variety of applications. The star sensor is a high-precision attitude measurement instrument; therefore, determining how to validate its accuracy is critical in guaranteeing its practical precision. The dynamic precision evaluation of the star sensor is more difficult than a static precision evaluation because of dynamic reference values and other impacts. This paper proposes a dynamic precision verification method of star sensor with the aid of inertial navigation device to realize real-time attitude accuracy measurement. Based on the gold-standard reference generated by the star simulator, the altitude and azimuth angle errors of the star sensor are calculated for evaluation criteria. With the goal of diminishing the impacts of factors such as the sensors’ drift and devices, the innovative aspect of this method is to employ static accuracy for comparison. If the dynamic results are as good as the static results, which have accuracy comparable to the single star sensor’s precision, the practical precision of the star sensor is sufficiently high to meet the requirements of the system specification. The experiments demonstrate the feasibility and effectiveness of the proposed method.

## Introduction

The inertial navigation system (INS), which can provide continuous and comprehensive navigation information of a carrier by using a gyro and an accelerometer, has been widely applied in military and civilian fields^1–3^. However, navigation errors accumulate over time in this system as a result of many error sources, such as initialization error, inertial sensor bias and computational error. Therefore, other navigation approaches are necessary to mitigate these errors in INS^4–8^. With the development of optoelectronics and image-processing techniques, the star sensor based on a charge-coupled device (CCD) or complementary metal-oxide semiconductor (CMOS) has become an attitude measurement instrument with the highest accuracy^9–11^. The star sensor offers many good characteristics: it is non-radiating, invulnerable to jamming and invariant to changes in time and distance, and it can obtain high-precision attitude information of a body in an inertial frame^12, 13^. Therefore, the stellar-inertial navigation system combines INS and the star sensor to take advantage of both their merits; indeed, the stellar-inertial navigation system is a promising combination for application to marine systems, military aircraft and deep-space exploration^14–18^. Because the accuracy of the star sensor, which can reach the arc-second level, directly determines the integrated navigation accuracy, verification of star sensor accuracy becomes an important step in stellar-inertial integration applications.

Many researchers have studied calibration and accuracy verification methods for the single star sensor^19–23^. The standard procedure is that the accuracy of the star sensor is tested after the application of various calibration methods; in other words, the verification experiment aims to verify the presented calibration method and then demonstrates the accuracy of the star sensor. Various calibration algorithms, both ground-based and on-orbit, have been proposed to estimate the most effective values of the optical parameters of the star camera using least-squares estimation or another fitting method^24–27^. Then, laboratory simulations or real night-sky tests are implemented to evaluate the accuracy of the star sensor after parameter compensation and to further assess the performance of the calibration methods. Thomas developed a complete calibration and qualification process for the TERMA star tracker to test its performance^28^. In addition, with the advanced development of the high-precision star simulator, the in-lab calibration accuracy of the single star sensor has improved dramatically^29, 30^. Regarding the accuracy evaluation method of the star sensor, Sun proposed an accuracy measurement method for a star tracker based on the direct astronomical observation under real night-sky conditions, taking the precise motion of the Earth as the reference^31^. Some real night-sky experiments are also conducted using a telescope to evaluate the actual accuracy of the star sensor^32^.

Due to many influencing factors, such as environment, mechanism and temperature, the star sensor accuracy in the integrated system also needs to be verified. The accuracy of the single star sensor provided by the previous calibration procedure cannot be the only basis for this verification because the star sensor is set for a period of time after in-lab calibration. Currently, there is not sufficient data in the literature to study accuracy verification methods for the star sensor in the stellar-inertial integrated system. Some scholars presented a calibration method for the stellar-inertial integrated system to improve the integrated precision^33, 34^. A novel alignment and calibration method was proposed, which combines inertial and stellar observations using an extended Kalman filter algorithm^35^. Some online autonomous calibration methods of integrated stellar-inertial navigation have also been proposed, with some maneuvers necessary to calibrate the inertial measurement unit (IMU) bias and star sensor installation errors^36, 37^. However, their precondition is that the star sensor performed well. Therefore, it is essential to verify its accuracy in the stellar-inertial integrated system.

When the star sensor is applied to a dynamic work environment, such as for missile-borne applications, the dynamic accuracy also must be guaranteed to capture stars effectively^38^. The existed in-lab methods for the star sensor accuracy measurement are under static condition, besides night sky experiments are under quasi-static condition (15 deg/h). There are no existed methods to assess dynamic accuracy of star sensor at every point in dynamic process. Focusing on the star sensor in the stellar-inertial integrated system, this paper proposes a dynamic precision verification method of star sensor with the aid of inertial navigation device to realize real-time attitude accuracy measurement. Utilizing the gold-standard reference which is the azimuth and elevation angles of star vector in navigation coordinate frame provided by star simulator, the altitude and azimuth angle errors of the star sensor are calculated for use in the evaluation criterion. A novel point is that the static and dynamic precision under the same trajectory and conditions are compared at the same time with the accuracy of the single star sensor. The experiments demonstrate that the proposed verification procedure is feasible and effective in practical applications.

## Methods

**Figure 1:**
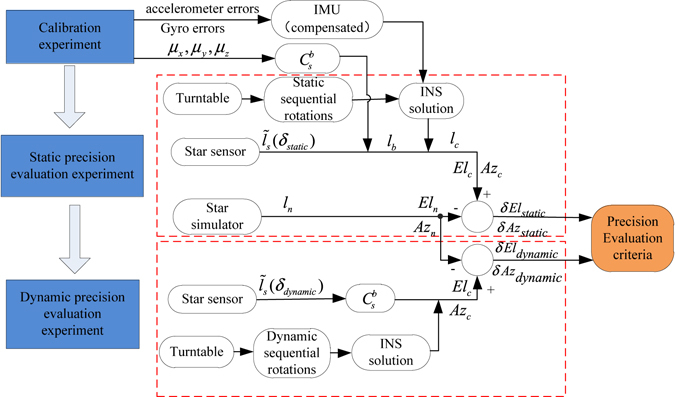
The precision-evaluation framework of the proposed method.

The specific process of the proposed precision evaluation method is divided into three steps: first, to improve the effectiveness of the verification process, the calibration for the stellar-inertial integration system is performed to confirm afresh all parameters for compensation in subsequent verification steps. Second, the static accuracy verification of the star sensor, for comparison with the dynamic accuracy verification results, is conducted. Third, the dynamic accuracy verification test of the star sensor is conducted to evaluate its dynamic precision. The framework of the whole process of the proposed precision evaluation method is presented in Fig. 1.

### Coordinate frames used in the paper

1. The local geodesic frame (*Ox*_*n*_ *y*_*n*_ *z*_*n*_) is the local-level coordinate frame located at the experiment position. A geographic east-north-upward (E-N-U) navigation frame is selected as the reference frame.
2. The system’s body frame (*Ox*_*b*_ *y*_*b*_ *z*_*b*_) is rigidly attached to the body-carrying navigation system. The y-axis lies along the system’s longitudinal axis, the z-axis points upward, and the x-axis completes the right-handed system.
3. The star sensor frame (*Ox*_*s*_ *y*_*s*_ *z*_*s*_) has its origin at the center of the image plane of the star sensor with the optical axis (y-axis) pointing toward a star.
4. The computed navigation coordinate frame (*Ox*_*c*_ *y*_*c*_ *z*_*c*_) is the local-level coordinate frame located at the computed position.

### The transformation matrix from the star sensor coordinate frame to the geographic coordinate frame

**Figure 2:**
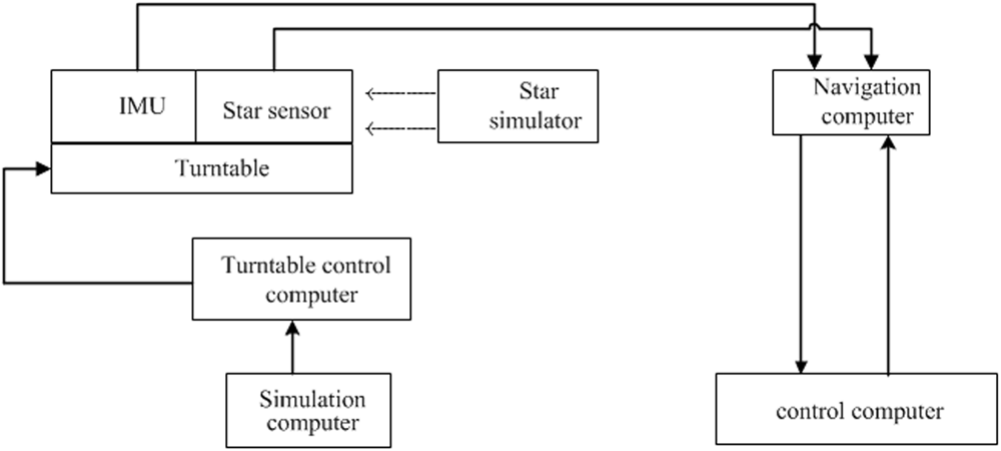
The experimental principle of calibration for the stellar-inertial integrated system.

The proposed precision evaluation method employs the information generated from the star simulator, which is a high-precision reference value in the geographic coordinate frame, as the gold-standard reference. It is necessary to transform the starlight vector measured by the star sensor into the geographic coordinate frame. The transformation matrix from the measurement frame to the geographic frame can be obtained by the INS navigation solution using information from the gyro and accelerometer. Then, the transformation matrix from the body frame to the star sensor frame is also calculated. As a result, to improve the reliability of the proposed method, the first step is the precise calibration of the inertial sensor and installation errors between the star sensor and the body frame; the work principle of this process is shown in Fig. 2. This process is first conducted to confirm the error parameters of the sensors in the stellar-inertial navigation system.

### The static precision evaluation of the star sensor

A high-precision star sensor, theoretically, has the same accuracy in a single instrument as a stellar-inertial navigation system. In addition, the static and dynamic precision in the integrated system should be comparable. Therefore, the static precision evaluation of the star sensor is implemented for comparison; the principal procedure is given in the following description.

In the proposed method, the gold-standard reference and the starlight vector measured by the star sensor are compared to calculate the errors. Obtaining the corresponding measurement information of celestial angles is the main objective of this integration algorithm. The differences in azimuth and elevation angles between the gold-standard reference and measurement by the star sensor are used as precision evaluation criteria, which can be written as

1 $${\delta }{El}=E{l}_{c}-E{l}_{n}\,{\delta }\mathrm{Az}=A{z}_{c}-A{z}_{n}.$$

where *Az*_*n*_ and *El*_*n*_ are the azimuth and elevation angles in the geographic coordinate frame, respectively, *Az*_*c*_ and *El*_*c*_ are the azimuth and elevation angles calculated by star sensor in the computed navigation coordinate frame, respectively. They can be obtained by following steps.

**Figure 3:**
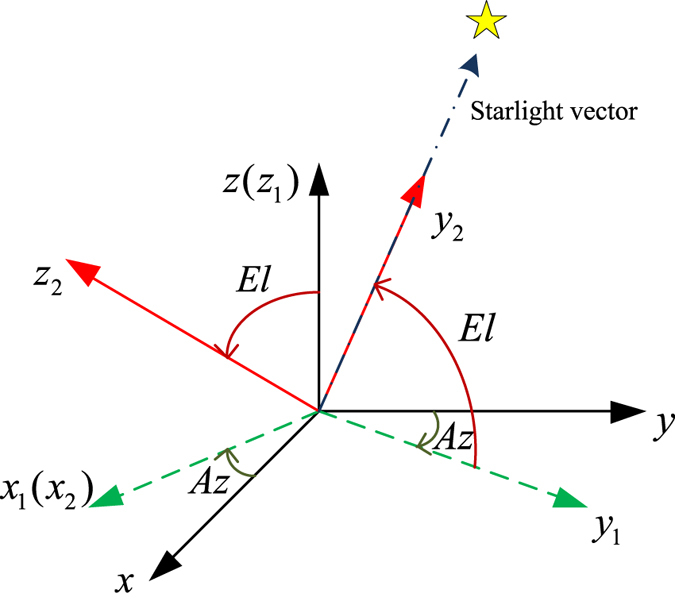
The definition of the azimuth and elevation angles.
  The relationship between the starlight vector in the reference frame and the azimuth and elevation angles is written as
  3 $$l=[\begin{array}{c}\sin \,Az\,\cos \,El\\ \cos \,Az\,\cos \,El\\ \sin \,El\end{array}].$$
  Then, the azimuth and elevation angles in the geographic coordinate frame can be calculated by
  4 $$\begin{array}{c}E{l}_{n}={\sin }^{-1}[{l}_{n}(3)]\\ A{z}_{n}={\tan }^{-1}[\frac{{l}_{n}(1)}{{l}_{n}(2)}].\end{array}$$
- (2) Then, the *El*_*c*_ and *Az*_*c*_ can be calculated by the starlight vector in the computed navigation coordinate frame.
  5 $$\begin{array}{c}E{l}_{c}={\sin }^{-1}[{l}_{c}(3)]\\ A{z}_{c}={\tan }^{-1}[\frac{{l}_{c}(1)}{{l}_{c}(2)}].\end{array}$$
- (3) The starlight vector in the computed navigation coordinate frame can be calculated by
  6 $${l}_{c}={\tilde{C}}_{b}^{n}{l}_{b}=[I-(\varphi \times )]{C}_{b}^{n}{l}_{b}.$$
  In this equation, *l*_*b*_ is the starlight vector in the body frame. INS can produce the attitude matrix ${\tilde{C}}_{b}^{n}$ by navigation solution, but it contains attitude errors when compared with the true attitude matrix ${C}_{b}^{n}$.
  7 $${\tilde{C}}_{b}^{n}=[I-(\varphi \times )]{C}_{b}^{n}$$
  where *ϕ* is the attitude error.
- (4) The star sensor can detect the line of sight $${\tilde{l}}_{s}=[\begin{array}{ccc}{x}_{s} & {y}_{s} & {z}_{s}\end{array}]$$ from the star simulator, which contains static measurement error *δ*_*static*_. Then the starlight vector in the body frame can be expressed as
  8 $${l}_{b}={C}_{s}^{b}{\tilde{l}}_{s},$$
  where ${C}_{s}^{b}$ is the transformation matrix from the star-sensor frame to the body frame. In the first calibration step, three installation errors of the star sensor $$[\begin{array}{ccc}{\mu }_{x} & {\mu }_{y} & {\mu }_{z}\end{array}]$$ are estimated. Then, they are used to compute the transformation matrix ${C}_{s}^{b}$, which can be written as
  9 $${C}_{s}^{b}=[\begin{array}{ccc}\cos \,{\mu }_{y}\,\cos \,{\mu }_{z}-\,\sin \,{\mu }_{y}\,\sin \,{\mu }_{x}\,\sin \,{\mu }_{z} & \cos \,{\mu }_{y}\,\sin \,{\mu }_{z}+\,\sin \,{\mu }_{y}\,\sin \,{\mu }_{x}\,\cos \,{\mu }_{z} & -\sin \,{\mu }_{y}\,\cos \,{\mu }_{x}\\ -\cos \,{\mu }_{x}\,\sin \,{\mu }_{z} & \cos \,{\mu }_{x}\,\cos \,{\mu }_{z} & \sin \,{\mu }_{x}\\ \sin \,{\mu }_{y}\,\cos \,{\mu }_{z}+\,\cos \,{\mu }_{y}\,\sin \,{\mu }_{x}\,\sin \,{\mu }_{z} & \sin \,{\mu }_{y}\,\sin \,{\mu }_{z}-\,\cos \,{\mu }_{y}\,\sin \,{\mu }_{x}\,\cos \,{\mu }_{z} & \cos \,{\mu }_{y}\,\cos \,{\mu }_{x}\end{array}].$$
- (5) As a result, the starlight vector in the computed navigation coordinate frame^37^ can be written as

- (1) The star simulator, which is mounted at a known position with precise orientation, can generate the gold-standard reference of the starlight vector in the geographic coordinate frame.
  2 $${l}_{n}=[\begin{array}{ccc}{x}_{n} & {y}_{n} & {z}_{n}\end{array}]$$
  The starlight vector^39^ is defined in terms of azimuth *Az* and elevation *El* relative to a reference frame (*O*−*xyz*), as shown in Fig. 3. The first rotation is about the z-axis through the *Az* angle, resulting in an intermediate frame *O*−*x*_1_ *y*_1_ *z*_1_. Then, a rotation about *x*_1_ through the *El* angle is performed to establish the starlight-vector axis in frame *O*−*x*_2_ *y*_2_ *z*_2_.

10 $${l}_{c}={\tilde{C}}_{b}^{n}{l}_{b}=[I-(\varphi \times )]{C}_{b}^{n}{C}_{s}^{b}{\tilde{l}}_{s}.$$

It is seen from equation (10) that the influencing factors contributing to errors in *l*_*c*_ include attitude matrix error, installation errors of the star sensor and measurement error of the star sensor.

The measurement error of the star sensor *δ*_*static*_ is what the proposed precision evaluation method will calculate.

The impact of installation errors on the star sensor, $$[\begin{array}{ccc}{\mu }_{x} & {\mu }_{y} & {\mu }_{z}\end{array}]$$, has been decreased by the first calibration step and subsequent compensation.

The attitude matrix error of the inertial navigation solution can be written as

11 $$\dot{\varphi }=\delta {\omega }_{in}^{n}+\varphi \times {\omega }_{in}^{n}-\delta {\omega }_{ib}^{n}.$$

where ${\omega }_{in}^{n}={\omega }_{ie}^{n}+{\omega }_{en}^{n}$, ${\omega }_{ie}^{n}$ is the Earth-rate angular rotation vector in the navigation frame, ${\omega }_{en}^{n}$ is the navigation-to-Earth angular rotation vector in the navigation frame, and $\delta {\omega }_{ib}^{n}$ is the output error of the gyro.

According to the error propagation properties of the pure inertial navigation in short time^40^, attitude errors are caused by three factors.

(a) Initial attitude errors *ϕ*_0_

Initial attitude errors lead to attitude errors in the navigation solution because the calculation of inertial navigation is an integration process. Thus, in the proposed precision evaluation method, the initial attitude error is minimized by precise initial alignment using a turntable.

(b) Gyro errors

The mathematical error model of three gyros generally can be expressed by

12 $$[\begin{array}{c}\delta {\omega }_{x}\\ \delta {\omega }_{y}\\ \delta {\omega }_{z}\end{array}]=[\begin{array}{ccc}\delta {S}_{x} & {E}_{xy} & {E}_{xz}\\ {E}_{yx} & \delta {S}_{y} & {E}_{yz}\\ {E}_{zx} & {E}_{zy} & \delta {S}_{z}\end{array}]\,[\begin{array}{c}{\omega }_{x}\\ {\omega }_{y}\\ {\omega }_{z}\end{array}]+[\begin{array}{c}{\varepsilon }_{x}\\ {\varepsilon }_{y}\\ {\varepsilon }_{z}\end{array}]+[\begin{array}{c}{w}_{gx}\\ {w}_{gy}\\ {w}_{gz}\end{array}],$$

where *δω*_*i*_ is the output error of the gyro in the *i*-axis, *i* = *x*, *y*, *z*, *ω*_*i*_ is the input rotation rate of the gyro in the *i*-axis, *δS*_*i*_ is the scale-factor error of the gyro in the *i-*axis, *E*_*ij*_ is the misalignment of the gyro in the *j*-axis relative to the gyro in the *i*-axis, *ε*_*i*_ is the bias of the gyro in the *i*-axis, and *w*_*gi*_ is the random noise in the *i*-axis.

The gyro errors cause periodic oscillations of the attitude error. In an attempt to reduce this error source, precise calibration is performed to determine all constant errors, including bias, scale-factor errors and misalignments. Then, the results of the calibration are used to compensate outputs of gyros in real time.

(c) Accelerometer errors

Similarly, the mathematical error model of accelerometers can be given by

13 $$[\begin{array}{c}\delta {f}_{x}\\ \delta {f}_{y}\\ \delta {f}_{z}\end{array}]=[\begin{array}{ccc}\delta {K}_{x} & {M}_{xy} & {M}_{xz}\\ {M}_{yx} & \delta {K}_{y} & {M}_{yz}\\ {M}_{zx} & {M}_{zy} & \delta {K}_{z}\end{array}]\,[\begin{array}{c}{a}_{x}\\ {a}_{y}\\ {a}_{z}\end{array}]+[\begin{array}{c}{\nabla }_{x}\\ {\nabla }_{y}\\ {\nabla }_{z}\end{array}]+[\begin{array}{c}{w}_{ax}\\ {w}_{ay}\\ {w}_{az}\end{array}],$$

where *δf*_*i*_ is the output error of the accelerometer in the *i*-axis,*i* = *x*, *y*, *z*, *a*_*i*_ is the input acceleration rate in the *i*-axis, *δK*_*i*_ is the scale-factor error of the accelerometer in the *i*-axis, *M*_*ij*_ is the misalignment of the accelerometer in the *j*-axis relative to the accelerometer in the *i*-axis, ∇_*i*_ is the bias of the accelerometer in the *i* axis, and *w*_*ai*_ is the random noise in the *i*-axis.

The accelerometer errors can cause velocity errors and sequentially determine the ${\omega }_{en}^{n}$ error. It is same with gyro calibration: the calibration constant errors of the accelerometer are used to remove output errors.

Through the above error analysis, it is determined that the error sources impacting the measured starlight vector information on the navigation system are the star sensor measurement error and random noise after the first calibration step and initially precise alignment.

### The dynamic precision evaluation of the star sensor

In static precision evaluation, the star sensor captures the line of sight when the system is stationary. In practical applications, the stellar-inertial integrated system sometimes requires measurement information from the star sensor under in-motion conditions. Therefore, the dynamic precision evaluation of the star sensor is also necessary to verify its dynamic measurement precision in a dynamic application environment. The principle of dynamic precision evaluation is the same as that of static precision evaluation. The output error of the star sensor, which contains dynamic measurement errors *δ*_*dynamic*_, can be evaluated.

## Results

An experiment in the laboratory is conducted to validate the feasibility of the proposed method. The stellar-inertial navigation system under investigation includes a star sensor and an IMU consisting of three ring-laser gyroscopes (RLGs) and three quartz flexible accelerometers (QFAs). The update rate of the IMU is 100 Hz. The star sensor is mounted parallel to the body coordinate frame and the optical axis is aligned with the y body axis. The accuracy of the star sensor is 3″ (3*σ*)in the star sensor coordinate frame and its update rate is 10 Hz. The two most important pieces of equipment are the star simulator and three-axis turntable. The three-axis turntable is able to rotate the integrated system to different positions with high accuracy. Three star simulators are mounted in the east, north, and upward directions of the turntable. The parameters and accuracy of the star simulator are as follows: the star-pointing accuracy is 0.5″(3*σ*); it can simulate the star magnitude of 1; and the field of view is 6° × 6°. When the star sensor is rotated to align the optical axis with the star simulator, the bright light is measured to generate starlight vectors.

According to the aforementioned outline of the proposed method, the precision evaluation experiment on the star sensor is divided into three steps.

### Step 1: Calibration

The starlight vectors measured by the star sensor need to be transformed into the geographic coordinate frame to be compared with the gold-standard reference. Moreover, the transformation of the attitude matrix is provided by INS. Therefore, the calibration of the stellar-inertial integrated system is first implemented to determine the installation errors of the star sensor and the constant errors of the IMU, to further improve the precision evaluation performance. To increase the system observability, a sequential 10-position rotation is designed for the stellar-inertial navigation system to calibrate all sensor errors. The calibration trajectory is given in Table 1. Xg, Yg, and Zg represent east, north, and up in the local geographic coordinate frame.

**Table 1:** The designed 10-position rotations of the calibration trajectory.

Number	Attitude before rotation			Rotation angle(°)/rotation axis
	y	z	x	
1	Xg	Yg	Zg	+90/Y
2	Xg	Zg	−Yg	+90/Y
3	Xg	−Yg	−Zg	+90/Y
4	Xg	−Zg	Yg	+90/Y
5	Xg	Yg	Zg	+90/Z
6	−Zg	Yg	Xg	+90/X
7	Yg	Zg	Xg	+90/X
8	Zg	−Yg	Xg	+180/X
9	−Zg	Yg	Xg	+90/Z
10	−Xg	Yg	−Zg	+180/Z

Based on the observability analysis, the calibration trajectory can estimate bias, scale-factor error and misalignments of IMU and installation errors of the star sensor. Table 2 presents the final estimated values of all sensor errors.

**Table 2:** Estimated results of IMU and the star sensor.

Accelerometer error											
Bias (μg)			Scale factor (ppm)			Misalignments (/″)					
∇_*x*_	∇_*y*_	∇_*z*_	*δKx*	*δKy*	*δKz*	*M*_*xy*_	*M*_*xz*_	*M*_*yz*_	*M*_*yz*_	*M*_*zx*_	*M*_*zy*_
−46.7	−24.0	−40.1	1.5	−32.5	18.3	140.3	−287.3	−123.8	113.0	314.8	−215.7
**Gyroscope error**									**Star-sensor error**		
**Bias (°/h)**			**Scale factor (ppm)**			**Misalignments (/″)**			**Installation error (/″)**		
*ε*_*x*_	*ε*_*y*_	*ε*_*z*_	*δS*_*x*_	*δS*_*y*_	*δS*_*z*_	*E*_*xy*_	*E*_*xz*_	*E*_*yz*_	*μ*_*x*_	*μ*_*y*_	*μ*_*z*_
0.06	0.03	0.04	6.2	25.5	5.8	−453.7	−35.7	−415.5	195.2	160.7	83.7

As shown in the Table 1, the estimated biases of the gyros and accelerometers are (0.06°/h, 0.03°/h, 0.04°/h) and (−46.7*μg*, −24*μg*, −40.1*μg*), respectively. If they are not calibrated, the influence on the navigation solution is large; furthermore, the precision evaluation results of the star sensor are affected by other factors in addition to the measurement error of the star sensor. The three installation errors of the star sensor are 195.2″, 160.7″ and 83.7″, which are much larger than the accuracy of the star sensor, 3″. Thus, it is of great importance to calibrate and compensate for the installation errors of the star sensor.

In the navigation solution process, the outputs of the gyros and accelerometers are compensated for utilizing the estimates of sensor errors, thereby improving navigation accuracy. The transformation matrix is calculated using equation (5) to compensate for the star sensor output.

### Step 2: Static precision evaluation of the star sensor

The measurement precision of a single position is not sufficient to validate the performance of the star sensor. In a real situation, systems complete some motion and then utilize the star sensor to perform observations under stationary conditions. For this reason, a sequential 10-position rotation, given in Table 3, is designed to verify the static precision of the star sensor. The rotation rate is set as 9°/s and each position is maintained for 30 seconds. The entire process lasts 5 minutes. For the static test, the system is stationary for 20 seconds and the star sensor measures the line of sight in the last 5 seconds, and then it takes 10 seconds to rotate to the next position. In this experiment, the optical axis of the star sensor is the y-axis and three simulators are mounted in the east, north, and up directions, respectively. There are 7 positions at which the optical axis aligns with the star simulators, to use measurement information for precision evaluation. It is clear that the starlight vectors of the fifth position and the ninth position are pointed upward. According to the relationship between the starlight vector and celestial angles, it can be deduced that the azimuth angles at these two positions are arbitrary. Consequently, the azimuth angles at these two positions are not computed for precision evaluation. To prove the repeatability and stability, 6 groups of static experiments are performed.

**Table 3:** The designed 10-position rotations of the evaluation trajectory.

Number	Attitude before rotation			Rotation angle(°)/rotation axis
	x	y	z	
1	Zg	Xg	Yg	+90/Y
2	Yg	Xg	−Zg	−90/Z
3	−Xg	Yg	−Zg	−90/Z
4	−Yg	−Xg	−Zg	+90/X
5	−Yg	Zg	−Xg	+90/Z
6	−Zg	−Yg	−Xg	+90/X
7	−Zg	Xg	−Yg	+90/X
8	−Zg	Yg	Xg	+90/Z
9	Yg	Zg	Xg	−90/Z
10	Zg	−Yg	Xg	+90/X

Using accurate starlight vector information provided by the star simulator as the gold-standard reference, the azimuth and the elevation errors of star sensor measurement are computed and provided in Tables 4 and 5.

**Table 4:** The elevation angle errors of static precision evaluation.

Number	Extremum	Elevation angle errors at every position (/″)							3*σ*
		Position1	Position2	Position3	Position5	Position7	Position8	Position9	
1	Max	2.59	3.79	2.83	−0.26	2.06	2.49	−0.57	3.32
	Min	−2.25	−3.51	−3.14	−4.24	−2.75	−2.23	−3.95	
2	Max	3.32	2.91	3.72	−0.19	3.93	3.68	−0.87	3.58
	Min	−2.84	−3.58	−2.96	−3.84	−3.62	−4.31	−3.8	
3	Max	3.29	2.68	4.36	−0.37	3.58	3.91	−0.93	4.06
	Min	−2.91	−4.05	−3.62	−3.29	−2.84	−4.35	−3.25	
4	Max	3.61	3.92	2.88	−1.02	4.38	3.46	−0.39	4.13
	Min	−3.26	−2.68	−3.92	−3.99	−3.81	−4.05	−2.99	
5	Max	4.25	3.46	3.59	−0.98	2.86	3.48	−0.58	3.69
	Min	−3.31	−3.25	−4.12	−2.49	−3.53	−2.79	−4.34	
6	Max	4.23	4.05	3.51	−0.09	2.46	4.16	−0.19	3.83
	Min	−3.30	−3.52	−4.19	−4.32	−3.62	−3.08	−2.84

**Table 5:** The azimuth angle errors of static precision evaluation.

Number	Extremum	Azimuth angle errors at every position (/″)					3*σ*
		Position1	Position2	Position3	Position7	Position8	
1	Max	2.37	3.39	3.65	2.68	4.01	3.46
	Min	−2.99	−3.15	−2.35	−2.67	−3.58	
2	Max	4.29	2.94	3.79	3.94	3.61	3.57
	Min	−2.35	−3.51	−2.97	−3.65	−4.33	
3	Max	3.28	2.69	4.35	3.52	3.91	4.02
	Min	−2.91	−4.06	−3.63	−2.86	−4.35	
4	Max	3.65	3.54	2.85	4.32	2.94	4.26
	Min	−3.28	−4.21	−3.91	−3.86	−4.09	
5	Max	4.25	3.86	3.56	2.87	3.79	3.72
	Min	−3.32	−3.75	−4.10	−3.53	−3.84	
6	Max	3.56	2.98	3.52	2.57	3.48	3.91
	Min	−2.51	−3.53	−4.08	−3.72	−4.21	

Tables 4 and 5 give the positive maximum and negative maximum of celestial angle errors at every position of every group experiment. It can be seen that the azimuth and the elevation errors have good consistency and stability. The 3-standard-deviation values (3*σ*) of the celestial angle errors are calculated using all data of all observation positions in every group experiment. There are 50 points at every position. The 3*σ* values of elevation errors in 6 groups of experiments are 3.32″, 3.58″, 4.06″, 4.23″, 3.69″, and 3.83″, and the 3*σ* values of azimuth errors in 6 groups of experiments are 3.46″, 3.57″, 4.02″, 4.26″, 3.72″, and 3.91″. Based on the experimental results, it is concluded that the static precision of the star sensor is approximately 4″, which is comparable to that of the single star sensor, 3″. This result can be explained by the fact that the calculation is still affected by the small residual errors of IMU and the experimental environment noise, according to the above error analysis. In summary, the static precision evaluation results of the star sensor in the stellar-inertial integrated system prove that the static accuracy of the star sensor in experiments is high.

### Step 3: Dynamic precision evaluation of the star sensor

**Figure 4:**
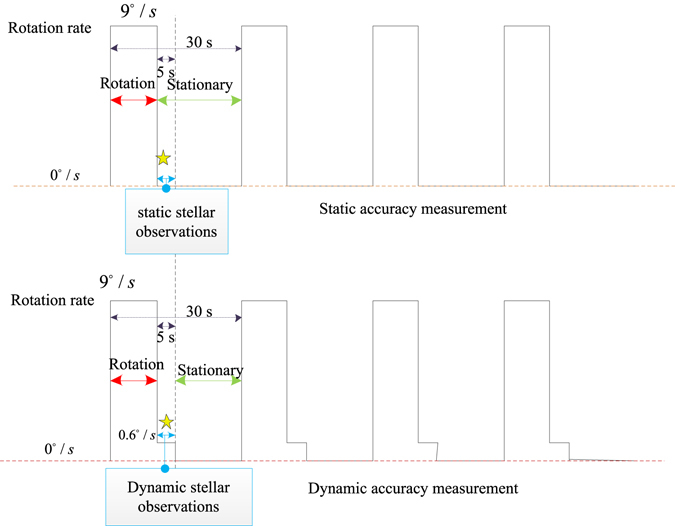
Comparison between static and dynamic experiments.

The dynamic tests employ the same rotation trajectory as the static tests for comparison, as shown in Fig. 4. The difference is that the static accuracy measurement experiment measures the line of sight under stationary conditions, whereas the dynamic accuracy measurement experiment measures the line of sight with a rotation rate of 0.6°/*s* in the last 5 seconds. Both experiments move from the present position to the next position with a rotation rate of 9°/*s*. Hence the precision of the star sensor is evaluated in motion at the same position as under the static conditions. Six groups of dynamic precision evaluation experiments are also conducted, and the results are presented in Table 6 and Table 7.

**Table 6:** The elevation errors of dynamic precision evaluation.

Number	Extremum	Elevation angle errors at every position (/″)							3*σ*
		Position1	Position2	Position3	Position5	Position7	Position8	Position9	
1	Max	4.17	3.47	5.05	−0.06	3.13	5.32	−0.05	4.96
	Min	−3.43	−4.41	−3.61	−5.76	−2.75	−6.79	−6.76	
2	Max	5.48	5.24	6.25	−0.09	4.53	5.62	−0.12	5.58
	Min	−4.25	−3.93	−5.64	−5.91	−3.19	−4.78	−6.35	
3	Max	6.02	4.53	5.32	−0.18	5.64	5.06	−0.26	5.12
	Min	−3.96	−6.25	−4.16	−5.71	−4.93	−6.35	−5.45	
4	Max	4.86	4.56	6.19	−0.54	4.96	4.33	−0.49	4.83
	Min	−3.87	−3.94	−4.58	−4.69	−3.06	−3.98	−4.95	
5	Max	3.89	5.46	6.49	−0.62	4.39	6.23	−0.18	5.82
	Min	−5.26	−5.09	−4.76	−6.16	−3.61	−5.76	−6.39	
6	Max	4.96	6.14	5.63	−0.54	3.91	6.51	−0.61	6.08
	Min	−5.73	−3.83	−6.46	−4.52	−3.46	−5.49	−5.18

**Table 7:** The azimuth errors of dynamic precision evaluation.

Number	Extremum	Azimuth angle errors at every position (/″)					3*σ*
		Position1	Position2	Position3	Position7	Position8	
1	Max	3.97	5.68	5.46	5.69	4.85	5.06
	Min	−4.89	−5.05	−4.01	−5.76	−3.99	
2	Max	4.28	6.16	6.18	5.46	5.94	5.67
	Min	−5.49	−4.86	−3.84	−4.15	−6.08	
3	Max	5.16	6.09	5.67	6.14	4.63	5.20
	Min	−3.59	−5.14	−3.91	−5.46	−4.82	
4	Max	4.29	5.49	5.16	6.08	4.38	4.98
	Min	−5.48	−5.06	−3.98	−5.32	−3.54	
5	Max	6.21	5.54	6.41	4.96	5.29	5.90
	Min	−5.48	−5.49	−5.67	−6.04	−4.69	
6	Max	5.42	6.14	4.57	6.59	5.64	6.13
	Min	−4.39	−5.42	−6.38	−5.18	−4.81	

From the given positive maximum and negative maximum of the celestial angle errors at every position of every group experiment, it can be concluded that the azimuth and the elevation errors also have good consistency and stability under motion. The 3*σ* values of the elevation errors in 6 groups of experiments are 4.96″, 5.58″, 5.12″, 4.83″, 5.82″, and 6.08″, and the 3*σ* values of the azimuth errors in 6 groups of experiments are 5.06″, 5.67″, 5.20″, 4.98″, 5.90″, and 6.13″. The experimental results show that the dynamic precision of the star sensor is approximately 6″ compared with the static precision of 4″, which proves the comparative accuracy. This result can be explained by the fact that the calculation is still affected by the small residual errors of IMU and dynamic noise according to the above error analysis. In sum, the dynamic precision evaluation results of the star sensor in the stellar-inertial integrated system prove that the dynamic accuracy of the star sensor in the experiment is as high as the static precision.

## Discussion

**Figure 5:**
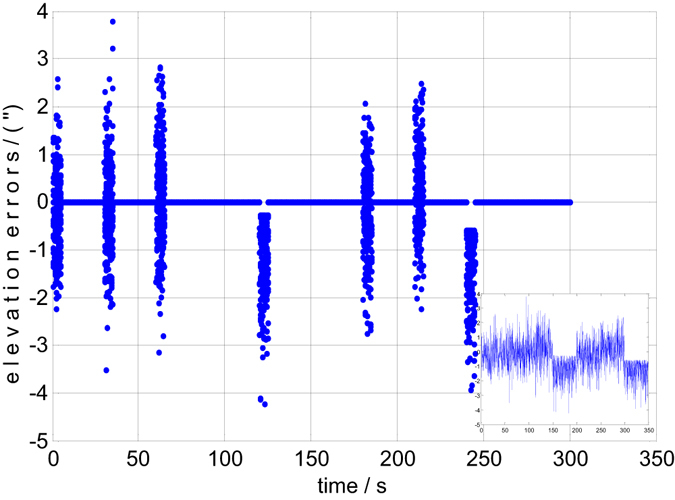
The static elevation errors of group 1.

**Figure 6:**
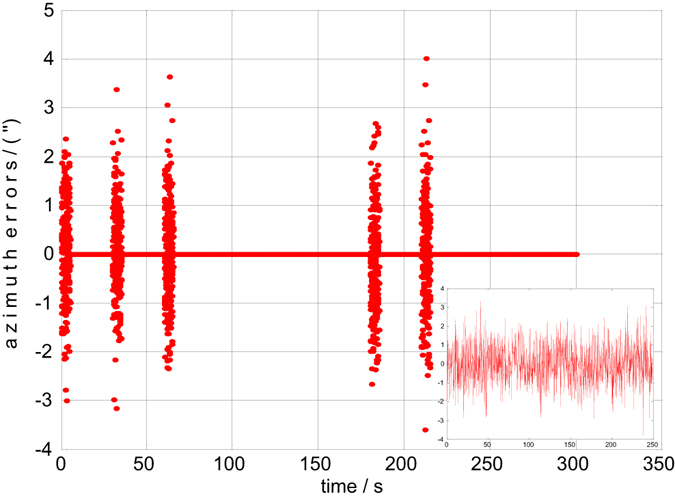
The static azimuth errors of group 1.

**Figure 7:**
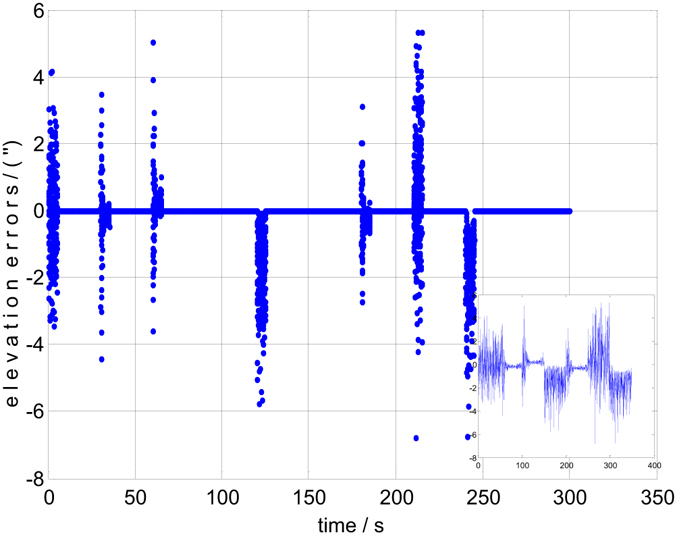
The dynamic elevation errors of group 1.

**Figure 8:**
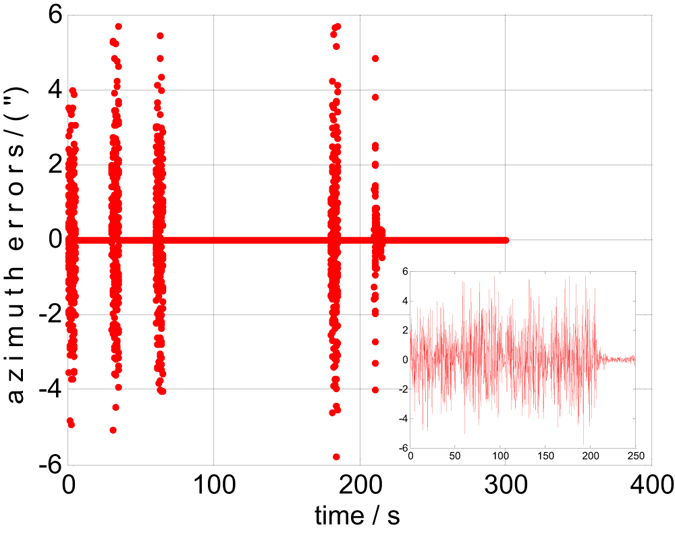
The dynamic azimuth errors of group 1.

Both the static and dynamic precision evaluation experiments are implemented in this section. The precision evaluation results for one of the 6 groups are depicted in Figs 5, 6, 7 and 8. There are 50 points of celestial angle error for 5 seconds at every position. It is clearly shown that dynamic and static celestial angle errors have consistent characteristics at every position. In addition, the dynamic error is only slightly larger than the static error. From the results of all 6 groups, it is determined that the static precision of the star sensor is approximately 4″ and the dynamic precision is approximately 6″. They are slightly larger than accuracy of the single star sensor of 3″ due to residual IMU errors and noise, according to the above error analysis, but they have the same order of magnitude, from which the conclusion can be drawn that the precision of the star sensor in the stellar-navigation integrated system is high. It is reasonable that the dynamic measurement error *δ*_*dynamic*_ is larger than the static measurement error *δ*_*static*_ because of the image motion question under dynamic situations.

The results of the experiment show the practicability and feasibility of the proposed method in practice, whose main advantages are listed as follows:

- The existing method for precision evaluation of the star sensor is aimed at the instrument level. However, when the star sensor is applied to the stellar-inertial integrated system, evaluating its performance in the integrated system is essential to guarantee sufficient precision in the navigation process.
- The proposed method first calibrated all sensor errors to be used for compensation in the subsequent precision evaluation experiment, which greatly diminished the influence of IMU errors and installation errors of the star sensor on the experimental results.
- The dynamic precision evaluation for the star sensor utilized the static precision results for comparison under the same trajectory condition. Because the proposed method employed the inertial navigation results, the static and dynamic precision evaluations are performed under the same environment and condition. Moreover, IMU outputs are compensated for using the same parameters.

There is a note that the proposed method is effective for any dynamic situation. In the experiments, the rotation rate is 0.6°/*s* due to that the maximum dynamic range of the used star sensor is 0.6°/*s*. Beyond this value, the precision of star sensor will decrease. In the practical application, the rotation rate can be any value if the dynamic range of the star sensor is high.

In the practical application of the proposed method, if the dynamic precision evaluation result is the same order of magnitude as the static results, whose precision is comparable to the instrument-level accuracy of the star sensor, it demonstrates that the precision of the star sensor in the stellar-inertial integrated system is sufficiently high for navigation. By contrast, the limitation of the proposed method is that the dynamic accuracy of the star sensor cannot be declared poor if the precision evaluation results are considerably larger than the instrument-level accuracy of the star sensor. The existing problems need to be determined from the whole stellar-inertial integrated system.

## Conclusions

The stellar-inertial navigation system has gained popularity in navigation applications such as airborne systems and missiles. In this paper, a dynamic precision evaluation method for the star sensor in the stellar-inertial integrated system is proposed. The star vector measured by the star sensor is transformed into the navigation frame through the attitude transformation matrix provided by the inertial navigation solution, whose accuracy is enhanced by first-step calibration for sensor errors. Utilizing the information of the star simulators as the gold-standard reference, elevation and azimuth angle errors in the geographic frame are calculated as precision evaluation criteria. Moreover, the static precision evaluation of the star sensor is also performed under the same evaluation trajectory condition with dynamic experiments for comparison. Regarding the star sensor used in the experiments, its static and dynamic precisions in the stellar-inertial integrated system are approximately 4″ and 6″, respectively, compared to the accuracy of the single star sensor of 3″. The analysis of static and dynamic experiments shows the feasibility and stability of the proposed method in actual practice, which can be used for evaluating the arc-second-level star sensor.
